# Cognitive Dissonance in Technology Adoption: A Study of Smart Home Users

**DOI:** 10.1007/s10796-020-10042-3

**Published:** 2020-07-25

**Authors:** Davit Marikyan, Savvas Papagiannidis, Eleftherios Alamanos

**Affiliations:** Newcastle University Business School, 5 Barrack Road, Newcastle upon Tyne, NE1 4SE UK

**Keywords:** Digitalisation, Cognitive dissonance, Coping mechanisms, Smart homes, Wellbeing

## Abstract

This study aims to address a research gap related to the outcomes of the use of technology when the performance falls short of initial expectations, and the coping mechanisms that users may deploy in such circumstances. By adopting Cognitive Dissonance Theory, the objectives of the study are a) to examine how dissonance, caused by the negative disconfirmation of expectations, may translate into a positive outcome and b) study how negative emotions, such as anger, guilt and regret, determine the selection of the mechanism to reduce dissonance. The theorised model was tested using a cross-sectional research design and a sample of 387 smart home users. The focus on smart home users fitted the objectives of the study due to the high expectations that users form and the challenges that the utilisation of technology sometimes causes. The collected data was analysed using structural equation modelling. Findings indicate that post-disconfirmation dissonance induces feelings of anger, guilt and regret, correlating with dissonance reduction mechanisms, which in turn have a distinctive effect on satisfaction and wellbeing. The findings of the study contribute to the discussion on expectation-disconfirmation and cognitive dissonance, by illustrating the interrelationship between emotional, cognitive and behavioural factors following the evaluation of technology performance and confirming that negative disconfirmation may result in satisfaction.

## Introduction

The ubiquitous embeddedness of intelligent objects (e.g. work and residential areas) is speeding up the transformational impact of digitalisation on society (Papagiannidis and Marikyan, 2019, Gupta et al., 2018). The utilisation of intelligent systems creates smart and data-rich environments, contributing to the societal sustainability due to the application of the big-data analytics ecosystem (Gupta et al., 2018). Big data and business analytics have become a new form of value creation and a source of sustainability solutions, accelerating economic, environmental and social growth (Pappas et al., 2018, Mikalef et al., 2019). For example, the application of intelligent systems and sensors (smart homes) in residential areas can potentially address societal needs and bring environmental and economic benefits (Marikyan et al., 2019, Raad and Yang, 2009, Baudier et al., 2018, Li et al., 2016) by creating energy-conscious and goal-oriented smart environments (Hussain et al., 2009, Palanca et al., 2018, Gupta et al., 2018). In addition, the data generated by intelligent devices, such as smart homes, can provide knowledge about the behaviour/interaction of users, suggesting solutions that may tackle social, environmental and economic challenges (Pappas et al., 2018). Companies may take advantage of data-driven knowledge to invest in innovations leading towards a sustainable society. Therefore, the utilisation of intelligent devices in homes can have significant social implications. However, the digital transformation of residential areas raises high user expectations (Dwivedi et al., 2019), which may undermine post-performance evaluation (Sun and Medaglia, 2019, Fan and Suh, 2014). Unmet beliefs about technology performance, in turn, inhibit the long-term utilisation of technology (Bhattacherjee, 2001). Hence, it is important to consider the psychological factors that the perception and experiences of the promised performance entail. This will help understand users’ behavioural patterns and facilitate the adoption of new technologies.

The literature provides useful insights into the cognition and behaviour of users after the evaluation of the performance of the technology. In Diffusion of Innovations Theory, the implementation of innovative technology may either end up with the confirmation or disconfirmation of an initial decision to adopt the technology. The decision is dependent on the perceived characteristics of the innovation, while the confirmation process reflects the degree to which technology performance produces perceptions consistent with prior beliefs (Rogers, 1995). Research in the expectation-(dis)confirmation domain has focused on confirmation (Gong et al., 2018) or the positive disconfirmation front, whereby technology outperforms initial expectations (e.g. with studies focusing on the correlation between positive disconfirmation and satisfaction) (Hsieh et al., 2010, McKinney et al., 2002). Still, the literature lacks insight into the psychological consequences following technology performance that does not match up to prior beliefs. Considering the importance of understanding the outcomes of negatively disconfirmed expectations, the focus of this research is to explore the behavioural and cognitive mechanisms users may resort to when the performance of new technology does not meet initial expectations.

There are three important gaps that need to be considered. Firstly following the expectation-disconfirmation and innovation diffusion perspectives, the negative disconfirmation of initial beliefs about technology performance is expected to result in dissatisfaction (Bhattacherjee, 2001, Bhattacherjee and Premkumar, 2004) and discontinuous use intention (Rogers, 1995, Huang et al., 2013). However, another perspective suggests that the negative disconfirmation may induce an affective state that reduces the perceived discrepancy between expectation and performance, thus potentially leading to satisfaction (Festinger, 1962, Harmon-Jones and Mills, 2019, Sparks et al., 2012). This suggests that the “disconfirmation-satisfaction” relationship is still under-researched. Secondly, the cognitive perspective on the outcomes of disconfirmation points to the complexity of the cognitive and behavioural processes that negative disconfirmation entails (Festinger, 1962). However, the literature does not offer any insights into the psychological factors and behavioural responses following disconfirmation. Exploration of those factors would explain the conditions in which satisfaction can be achieved. Previous research studies highlighted the role of situational factors in attenuating the strength of disconfirmation and dissatisfaction. Those factors include the magnitude of the discrepancy between perception and expectation (Oliver, 1980, Khurana, 2011), the importance of the outcome and the level of involvement with the product (Patterson, 1993), and the regulatory role of reputation on the formation of expectation and perception (Walsh et al., 2016). Such findings either illustrated the potential moderation effect on the disconfirmation-satisfaction relationship or investigated the factors decreasing the likelihood of negative disconfirmation. However, they did not provide an explanation of the behavioural and cognitive patterns of individuals experiencing the disconfirmation of expectations. Thirdly, prior research has postulated that affective states and psychological discomfort motivate users to adopt behaviours that reduce the perceived discrepancy between the two types of cognition (i.e. expectation and perceived performance) (Festinger, 1962). However, the relationship between the main types of emotions and cognitive/behavioural patterns of individuals have not been examined. Although past studies have examined negative emotions, such as anger, regret and guilt, they treated them as a single construct (Jean Tsang, 2019, Gosling et al., 2006). Still, if examined independently, each emotion may result in different behavioural responses (Beaudry and Pinsonneault, 2010). In addition, the findings on the effect of some types of emotions are conflicting in terms of their impact on approach and avoidance behaviours (Miller, 1977, Davvetas and Diamantopoulos, 2017).

Given the above gaps, the aim of this research is three-fold. First, it aims to find the correlation between negative disconfirmation and satisfaction. In line with this aim, the objective of the paper is to examine the post-performance dissonance arousal induced by the discrepancy between performance and expectations using cognitive dissonance theory. The theory serves as a framework for explaining the behaviour of people experiencing cognitive inconsistencies, such as the expectation-performance gap (Festinger, 1962). Second, the study aims to provide an understanding of the cognitive and behavioural patterns of individuals following disconfirmation. In line with the cognitive dissonance theory, this research explores potential strategies that individuals use to attenuate the negative feelings following unmet expectations. The adoption of the theoretical framework enables us to explore how behavioural and cognitive responses to negative disconfirmation relate to satisfaction. The third aim of the study is to shed light on the role of different types of emotions associated with dissonance in predicting particular dissonance reduction strategies. To address this aim, the paper examines the effect of anger, guilt and regret on reduction strategies, eliminating dissonance through cognitive or behavioural adjustments. The examination of different types of emotions makes it possible to explore their motivational role in inhibiting or facilitating the behaviour that causes psychological discomfort. The theorised research model is tested using smart home technology as a context of the study. Smart homes manifest the digitalisation of private environments using intelligent systems (GhaffarianHoseini et al., 2013). The technological characteristics, potential impacts, promised benefits (Demiris and Hensel, 2009, Demiris et al., 2008) and the challenges that the utilisation sometimes causes (Hargreaves et al., 2018, Nicholls et al., 2017, Strengers and Maller, 2011) make smart homes a good context fitting the objectives of the study.

The findings of the study add to the literature in three ways. First, the study contributes to the discussion on expectation-disconfirmation, by illustrating complex psychological processes following the evaluation of technology performance. The findings confirm that negative disconfirmation may result in satisfaction. Second, the study contributes to the cognitive dissonance literature by explaining the interrelation of emotional, cognitive and behavioural factors underpinning the reduction of dissonance. Third, the study provides evidence on the psychological factors affecting consumer experience with new technologies, which has been under-researched so far. In addition, the findings provide evidence about the consequences of smart homes utilisation following weak performance, which has been lacking to date.

The paper is structured as follows. First, the paper presents the literature review and hypotheses. This section discusses the literature on smart homes and technology adoption. Then, the theoretical framework is presented, followed by the discussion of the theoretical background supporting each proposed relationship in the model. Second, the paper explains the methodological processes undertaken to conduct the study. The next sections present the results of path analysis and a discussion of the findings. The paper concludes with a summary of the research, an outline of the limitations and future research suggestions.

## Literature Review and Hypotheses

### Smart Homes

Digitalisation has fuelled the development of a sustainable lifestyle by transforming traditional homes into smart ones. A smart home is defined as “*a residence equipped with computing and information technology, which anticipates and responds to the needs of the occupants, working to promote their comfort, convenience, security and entertainment through the management of technology within the home and connections to the world beyond*” (Aldrich, 2003). This definition describes smart homes in a comprehensive way, embracing all components, including their technological characteristics, services, functions and benefits. In terms of technology, smart homes consist of software and hardware, represented by physical objects, wearable devices and sensors, which are capable of monitoring and detecting changes in the environment and users’ body conditions, and respond accordingly (Arunvivek et al., 2015, Orwat et al., 2008). Modern smart homes are built on context-aware and intelligent systems, enabling the multi-connectivity of devices, real-time tracking and behaviour recognition. Artificial intelligence enables smart homes to gather data and build knowledge about users’ preferences by observing their behavioural patterns and to provide tailored responses (Lynggaard and Skouby, 2016, Khalid and Ah, 2015, Skouby et al., 2014). The configurations of home intelligent systems determine the functions and services, which can benefit users and society (Chan et al., 2009, Chan et al., 2008).

Smart homes offer five main types of services, namely support, monitoring, the delivery of therapy, the provision of comfort and consultancy (Chan et al., 2009, Alam et al., 2012, De Silva et al., 2012, De Silva and Darussalam, 2008). These services facilitate sustainable development and users’ wellbeing (Wong and Li, 2008) by addressing the environmental, social and economic needs of society (Li et al., 2016). In terms of environmental value, the utilisation of environment monitoring systems (e.g. smart lighting, gas, energy management) and smart home appliances (e.g. smart refrigerators, dishwashers, locks, doors) offer comfort, consultancy and monitoring services. Such devices automate household tasks and reduce energy usage by automatically adapting energy supply, providing feedback on consumption and offering recommendations on the efficient use of electricity (Arunvivek et al., 2015, Alam et al., 2012, Chan et al., 2008, De Silva et al., 2012). Social value is reflected in the promotion of the physical and psychological wellbeing of people in need through access to remote health therapy and virtual interaction, support in independent living, monitoring of health conditions and the provision of consultancy. Those services are possible by implementing remote alarms, robots and robotic devices for rehabilitation, telecare, drug delivery systems, voice recognition technology, the integration of sensors and wearable devices (Demiris, 2004, Alam et al., 2012, Patel et al., 2012, Ranasinghe et al., 2016, Peetoom et al., 2015, Rantz et al., 2005, Chan et al., 2009, Masuda et al., 2005). Economic value is achieved by transforming traditional healthcare to homecare and taking advantage of smart lighting and energy management systems, which enable users to reduce spending on resource consumption and physical visits to a doctor (Marikyan et al., 2019).

Smart home benefits determine the user segments of the technology (Wilson et al., 2014). For example, smart homes provide social connectivity, remote healthcare accessibility, they enable health monitoring and the prevention of health-threatening events (Reeder et al., 2013, Demiris and Hensel, 2009, Chan et al., 2009), which fits the requirements of elderly, vulnerable people and those in need of assistance (Cesta et al., 2011, Ehrenhard et al., 2014). The reduction of energy and water usage through smart systems brings financial efficiency (Balta-Ozkan et al., 2014a, Zhou et al., 2016), which is important for rational and price-conscious people in low- and middle-income households (Wilson et al., 2014). Also, the technical sophistication of smart homes and constant upgrades make the technology attractive for technology enthusiasts seeking constant ICT enhancement (Park et al., 2003).

Although smart homes promise benefits that can address the needs of wide user segments, the adoption of the technology is still low (Coskun et al., 2018, Marikyan et al., 2019). The adoption rate can be explained by perceived risks and challenges, which relate to technological, financial ethical and legal issues and knowledge gaps. Technologically, smart homes are not easy to use, control, maintain and integrate with other technologies (Balta-Ozkan et al., 2014b). Usability of smart homes is inhibited by the lack of knowledge about technology operation, which is often associated with resistance to change (Kerbler, 2013, Keith Edwards and Grinter, 2001, Balta-Ozkan et al., 2013b). The integration of smart homes in the household requires time for familiarisation and adaptation, which limits the use of technology (Hargreaves et al., 2018). The connectivity of devices through the internet raises privacy and security issues, which is of primary concern for some user groups (Balta-Ozkan et al., 2013a, Chan et al., 2009). In addition, the literature provides evidence indicating that expectations about the energy efficiency of smart home devices are sometimes not fulfilled (e.g. (Herrero et al., 2018, Hargreaves et al., 2018)). For example, the results of a longitudinal field trial showed little support for the argument that smart homes substantially reduce energy consumption. Instead, the use of smart homes facilitated more intensive use of energy (Nicholls et al., 2017, Hargreaves et al., 2018). Another observation showed that users tend to manually switch on/off energy management devices, which suggests that the actual reduction in energy consumption due to smart home technology utilisation is far less than declared (Strengers and Maller, 2011). These studies provide evidence on the expectation-performance gap, which may inhibit the wider adoption of the technology. However, smart home literature lacks insight into the behavioural consequences of users’ disconfirmed expectations. Hence, the following sections provide a review of the literature on technology adoption and discuss the Cognitive Dissonance Theory to understand users’ behavioural patterns when technology performance falls short of expectations. The review of technology adoption literature emphasises the importance of the confirmation of prior expectations in the long-term technology utilisation. The findings shed light on the outcomes of expectation-performance evaluation in relation to satisfaction. The adoption of Cognitive Dissonance Theory provides complementary insight by explaining psychological outcomes that disconfirmed expectations entail. Also, it suggests what behavioural and cognitive changes make people satisfied with the technology despite unexpectedly weak performance.

### Technology Adoption

The technology adoption literature in the post-disconfirmation domain mostly uses the perspectives of expectation-(dis)confirmation (Bhattacherjee, 2001, Bhattacherjee and Premkumar, 2004, Oliver, 1980) and innovation diffusion (Rogers, 1995, Huang et al., 2013) to explain the outcomes of technology utilisation. The Expectation Confirmation Model (ECM) is widely used for studying IS users’ continuance intention. It postulates that satisfaction and post-adoption behaviour is predicted by the degree to which pre-exposure expectations are confirmed by the post-exposure experience (Bhattacherjee, 2001, Bhattacherjee and Premkumar, 2004). The theory is rooted in the expectation-disconfirmation theory, which posits that better than expected outcomes lead to satisfaction, which, in turn, contribute to continuous use intention (Oliver, 1980). For example, it was found that the confirmation of expectations about the playfulness of the world wide web leads to satisfaction, which, in turn, contributes to the users’ intention to reuse websites (Lin et al., 2005). The expectation confirmation, along with perceived usefulness, perceived ease of use and usage cost affect users’ satisfaction and subsequent post-adoption behaviour (Zhou, 2011). When technology performance is better than an expected quality (positive disconfirmation), users are more likely to feel satisfied (Hsieh et al., 2010, McKinney et al., 2002). In contrast, negative disconfirmation of initial expectations undermines the intention to adopt technology (Venkatesh and Goyal, 2010). Moreover, high expectations about alternative technology and weak performance of existing technology predict users’ switching behaviour (Fan and Suh, 2014).

The second perspective in the IS adoption research is put forward by Innovation Diffusion Theory, which postulates that the adoption of innovation is contingent on the degree to which the characteristics of the innovation (i.e. relative advantage, compatibility, complexity, observability, triability) are confirmed after its utilisation. Users reappraise innovation attributes during the confirmation stage, so they could reconsider the decision to continuously use innovation (Rogers, 1995, Huang et al., 2013). For example, the results of prior studies demonstrate that Internet technology adoption is predicted by confirmed expectations about the characteristics of the Internet, such as compatibility, image, financial slack and relative advantage (Lee, 2004). The intention to continuously use RFID was found to be dependent on the confirmation of performance expectations following the initial technology utilisation (Alamgir Hossain and Quaddus, 2011). Also, e-commerce system adoption was found to be positively related to the expected relative advantage and compatibility (Alam et al., 2007). Given the above perspectives, the negative disconfirmation of initial beliefs about technology characteristics and performance is expected to result in dissatisfaction and discontinuous use intention. However, Cognitive Dissonance Theory provides a competing perspective, suggesting that the negative disconfirmation might initiate the reduction of perceived discrepancy between expectation and performance (Festinger, 1962), thus potentially leading to satisfaction. The rationale for, and the justification of, the proposed argument are provided in the following section.

### Cognitive Dissonance

Cognitive Dissonance Theory has been used in IS research to explain the behaviour of individuals when they experience disparity between pre-service and post-service perception of products’ performance (Park et al., 2015, Venkatesh and Goyal, 2010). The theory postulates that a state of dissonance is triggered when an individual has two or more contradictory cognitions (Festinger, 1962). Dissonance, induced by disconfirmed expectations, triggers the psychological state associated with negative emotions and discomfort. This affective state influences the motivation of individuals to resolve the aroused dissonance (Festinger, 1962, Sweeney et al., 2000). To reduce dissonance, individuals can undertake a number of measures. These measures can be categorised into three main types, namely attitude change, consonant information-seeking and behaviour change (Festinger, 1962). Attitude change is defined as the modification of initial expectations or the perception of performance (O’Neill, 2004, Festinger, 1962, Harmon-Jones and Harmon-Jones, 2007). Individuals’ preferences towards a specific choice is strengthened and alternatives are rejected, increasing the consonant state of mind. Attitude change represents the post-factum justification of the product purchase or the rationalisation of the product performance, aimed at maintaining the integrity of someone’s decisions and their outcomes (Stephens, 2017, E. Ashforth et al., 2007, Harmon-Jones and Harmon-Jones, 2007). The consonant information seeking mode occurs when individuals selectively search for reaffirming information about the decision through different channels, such as advertising (Liang, 2016) or word-of-mouth (Kim, 2011). Behaviour change represents the withdrawal of the behaviour causing dissonance (Festinger, 1962). This reduction strategy is an aversive measure to eliminate the possibility of negative outcomes occurring in the future (McGrath, 2017). For example, the negative experience might result in the cancellation of the use of a particular brand (Lindsey-Mullikin, 2003). Similarly, exposure to negative word-of-mouth can result in the discontinuation of the product/brand use (Kim, 2011). However, a behaviour change requires significant effort, which makes it a less documented strategy to reduce dissonance (McGrath, 2017).

**Fig. 1:**
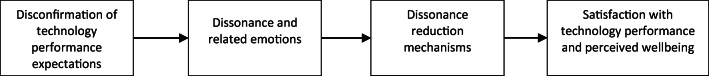
Overview of the conceptual model

Given the above we conceptualise the process users go through as a four-stage process (Fig. 1). First, the disconfirmation of technology performance vs initial expectations occurs; second, individuals start experiencing emotional discomfort; third, emotional discomfort induces behavioural or attitudinal actions to reduce dissonance; the fourth stage is the outcome of cognitive dissonance actions. In addition to testing the relationships among the above, the facilitating role of dissonance reduction in achieving satisfaction and perceived wellbeing is also tested.

### Hypothesis Elaboration

#### Disconfirmation of Technology Performance Expectations

Drawing on the Theory of Expectation-Confirmation, the individuals’ evaluation and satisfaction of the experience with technology is the result of the comparison of expectations and the performance (Bhattacherjee, 2001, Dai et al., 2015). Expectations refer to pre-exposure beliefs about a service or product (Susarla et al., 2003). The evaluation of pre-purchase expectations with actual performance can lead either to the confirmation or disconfirmation of the expectation. Confirmation results from the match between pre-exposure expectations and actual performance, while disconfirmation is the outcome of performance which is inconsistent with expectations. Disconfirmation is positive when actual experience with the use exceeds prior beliefs about the use. Negative disconfirmation occurs when performance falls short of expectations (Oliver, 1980, Kopalle and Lehmann, 2001). The inconsistency between the degree of perceived performance and prior beliefs represents the conflict of the two types of cognition, which can be explained by Cognitive Dissonance Theory. The inconsistency causes dissonance, which is associated with psychological discomfort (Festinger, 1962). The intensity of dissonance differs depending on the degree of discrepancy between initial cognition and the cognition after the exposure to technology use. The discrepancy can be small, falling within the zone of tolerance, without triggering dissonance arousal. As the magnitude of the discrepancy increases, the probability and the magnitude of dissonance arousal increases too (Szajna and Scamell, 1993). Dissonance can arise not only due to the discrepancy between expectation and performance, but the comparison of pre-service and post-service performance of technology or IS systems (Park et al., 2015). Pre-service performance may include the quality of pre-service customer service or website design. Post-service performance includes the evaluation of the object’s attributes related specifically to the use of the technology or IS system (Park et al., 2015). Based on the above, our first hypothesis is put forward:

- ***H1:*** *The disconfirmation of technology performance with prior expectations has a positive effect on dissonance arousal*

#### Dissonance and Related Emotions

Dissonance is associated with discomfort and uneasiness, which reflect negative emotions. The strength of emotions demonstrates the degree of dissonance arousal (Festinger, 1962). Past research has identified three emotions that can be associated with cognitive dissonance. The first is anger, occurring when people feel they are not responsible for the situation causing dissonance and/or are incapable of fulfilling the task (Harmon-Jones, 2004, Harmon-Jones et al., 2017). Anger is defined as a basic emotion, holding a number of other underlying similar, yet different emotions, like frustration, irritation or bitterness (Shaver et al., 1987). It has been reported that people who experience stronger cognitive dissonance have a stronger perception of anger and aggression (Soutar and Sweeney, 2003). The relationship between dissonance and anger explains the negative outcome of service performance and use of technology. For example, failure in technology performance raises anger and withdrawal behaviour, such as boycotting the retailer of the product (Donoghue and de Klerk, 2013). The use of technology contributes to the experience of anger and anxiety in situations when people have low self-efficacy in the use of computers (Wilfong, 2006). Self-efficacy represents the state when technology users feel incapable of realising the expected services (Bandura, 1977). Therefore, dissonance reflecting a disconfirmed belief about personal technical competence is more likely to be associated with anger.

The second emotion is guilt (Gosling et al., 2006, Turel, 2016). Guilt is associated with a feeling of shame and self-disappointment and can explain the psychological state between cognitive dissonance and the intention to discontinue the use of technology. Guilt is a response to the behaviour that causes moral dilemmas, such as the inconsistency with personal norms, values and self-standards (Harmon-Jones et al., 2017). Guilt can be experienced when a person feels responsible for the failure of technology performance, causing inconsistency with internal norms. The higher the control over the behaviour, the higher is the perception of guilt (Burnett and Lunsford, 1994). For example, IT addiction raises self-attributed negative emotion (i.e. guilt), which reflects the perception that a person is not capable of rationally utilising the technology and realising desired goals (Vaghefi and Qahri-Saremi, 2017). Other incidents with technology inducing guilt may include the excessive use of technology at the expense of important tasks (Turel et al., 2011) or ethical implications of the use of technology (Harrington, 1996).

The third emotion related to dissonance is regret (Roese and Summerville, 2005, Gilovich et al., 1995b). This is one of the negative outcomes of purchase decisions resulting in disconfirmed expectations (Oliver, 2014). Regret reflects self-blame for the behaviour that should not have been performed (Connolly and Zeelenberg, 2002, Gilovich et al., 1995b). Regret can be experienced when individuals choose a particular technology out of similar alternatives. In post-purchase situations, the strength of regret is conditioned by the degree to which non-selected alternatives represent the value for the individual. The experience of regret is stronger when the evaluation of the foregone alternative is increasing (Croyle and Cooper, 1983). For example, regret is experienced when the use of technology causes problems. Negative implications devalue the chosen technology and induce considerations about alternatives that could have been acquired instead (Dhir et al., 2016). Regret may occur not only as a result of issues with the utilisation of technology, but after the exposure to positive information about the services of an alternative technology (Kang et al., 2009). Also, individuals can feel regret when they realise that an alternative product could have been acquired at a lower cost (McConnell et al., 2000). Based on the above, the next hypothesis is proposed:

- ***H2:*** *Dissonance caused by the disconfirmation of technology performance with prior expectations has a positive effect on the arousal of a) anger, b) guilt and c) regret*

#### Dissonance Reduction Mechanisms

Emotions mediate the dissonance arousal and reduction processes (Festinger, 1962) because emotions are able to motivate and organise cognitions and actions (Izard, 2010). Emotions help interpret the signals of social interaction, communication and feeling states which underpin cognitive appraisals (Izard, 2010). Prior research has examined negative emotions in dissonance reduction as a unidimensional construct, embracing anger, fear, regret and anxiety (Jean Tsang, 2019, Gosling et al., 2006). However, this approach can be questioned given that emotions represent a complex process that guides people differently in various situations (Izard, 2010). Emotions can be differentiated by three aspects: a) affective valence, b) motivational direction and c) arousal. Affective valence refers to the degree to which people are positive or negative about the felt emotion and the state (Harmon-Jones et al., 2011, Harmon-Jones et al., 2017). Motivational direction refers to the role that the emotion plays in approach (behaviour aimed at reaching the goal) or avoidance (aversion from the goal achievement) behaviour (Harmon-Jones et al., 2017). The commitment to the behaviour by changing attitude and strengthening positive attitudes through the exposure to consonant information falls into approach behaviour. The lack of commitment, such as a change of behaviour as a result of dissonance, refers to withdrawal behaviour (Harmon-Jones, 2004). Arousal is the intensity of the feeling and psychological response to it (Harmon-Jones et al., 2017). In terms of affective valence, anger, guilt and regret refer to negative emotions. It is considered that negative emotions inhibit behaviour, which indicates withdrawal motivation (Harmon-Jones, 2004, Watson, 2000). However, when it comes to motivational direction, these types of emotions have a distinctive role in the cognitive dissonance strategies and the commitment to the behaviour causing dissonance (Harmon-Jones, 2004, Harmon-Jones et al., 2017). The distinctive motivational role of emotions is explained by different conditions in which emotions are manifested. The conditions include the degree of control over behaviour, the extent of responsibility for behavioural outcome, the justifiability of behaviour, the availability of better behavioural alternatives and the degree to which behaviour violates personal or social norms (Smith and Lazarus, 1993, Harmon-Jones et al., 2017, Harmon-Jones et al., 2003, Amodio et al., 2007, Connolly and Zeelenberg, 2002, Gilovich et al., 1995a).

Evidence suggests that anger resulting from the use of technology negatively affects its continuous use (Beaudry and Pinsonneault, 2010), which indicates the role of emotion in motivating avoidance behaviour. However, the findings on the motivational direction of anger are conflicting (Harmon-Jones, 2004, Harmon-Jones et al., 2004, Harmon-Jones et al., 2017, Carver, 2004). This inconsistency may be rooted in two reasons. First, the feeling of anger is often associated with other related emotions (e.g. irritation, shame, anxiety), motivating approach or avoidance behaviour. The interrelationship with other emotions affects the motivational direction of anger. For example, anger coupled with anxiety facilitates behaviour withdrawal (Harmon-Jones et al., 2017). Secondly, the motivational direction of anger depends on whether individuals feel responsible for the anger-inducing event and whether they have opportunities to undo the event. In situations of being intentionally harmed by another party, anger activates an approach-behaviour (Harmon-Jones et al., 2017). The common response in such situations is to punish the responsible party (Smith and Lazarus, 1993). However, the motivation to initiate any response is mitigated when there is no opportunity to ameliorate the situation causing anger (Harmon-Jones et al., 2003). Such anger is associated with feeling incapable of achieving the initial goal. It triggers the desire to change the goal orientation and switch to alternative options (Harmon-Jones, 2004, Carver, 2004). For example, anger is manifested when the use of technology inflicts security threats (Liang et al., 2019, Beaudry and Pinsonneault, 2010). When security threats occur, reduced commitment to technology and a subsequent behaviour withdrawal represent a defensive mechanism to avoid similar negative outcome in the future (Beaudry and Pinsonneault, 2010). Anger has a pro-active role in users’ behaviour, as it encourages individuals to seek out external means to cope with the emotion, which leads to the derogation of the behaviour causing anger (Liang et al., 2019). Also, it has been found that the failure of an appliance induces different levels of anger. The highest level of anger correlates with the intention to redress the experience and discontinue behaviour (Donoghue and de Klerk, 2013). As such, users who experience anger induced by an unexpected and unsatisfactory result of the use of technology are more likely to switch to another behaviour, rather than try to justify the negative outcome.

- ***H3:*** *Feeling anger negatively affects a) attitude change and b) consonant information search, and positively affects c) behaviour change*

Guilt is considered to be a self-regulatory emotion (Amodio et al., 2007). There are two theoretical perspectives on the role of guilt in motivating behaviour (Turel, 2016, Amodio et al., 2007, Harmon-Jones et al., 2017). There is evidence that guilt motivates avoidance mechanisms, namely, the discontinuation of technology usage (Turel, 2016). Another perspective postulates an opposite role of guilt in behaviour (Harmon-Jones et al., 2017, Amodio et al., 2007). In morally violating situations, people tend to look for the means to resolve guilt, which contributes to approach-motivational orientation (Harmon-Jones et al., 2017, Amodio et al., 2007). The cognitive dissonance reduction through attitude change and consonant information-seeking represent the means to resolve guilt. They reflect the way to justify an action retrospectively and continue the behaviour by subduing negative emotions (Ghingold, 1981b, Kelman, 1979). For example, people who engage in conversations to reduce psychological tension have a lower level of regret than people who do not try relief dissonance through communication (Stice, 1992). Such conversations represent a form of cognitive adjustment. Given the above, it is hypothesised that:

- ***H4:*** *Feeling guilt positively affects a) attitude change and b) consonant information search, and negatively affects c) behaviour change*

The literature provides evidence about the effect of regret on avoidance motivation (Gilovich et al., 1995b, Davvetas and Diamantopoulos, 2017). There are two reasons to suggest that regret has a negative effect on the continuous use of technology. The motivation for withdrawal behaviour stems from cognitive processes associated with regret, such as weak self-esteem and strong self-blame. Regret is a painful feeling, since it implies a personal fault in the negative outcome and raises counterfactual thinking (Connolly and Zeelenberg, 2002, Gilovich et al., 1995b). Counterfactual thinking refers to the ruminations about alternative decisions and potential consequences (Roese, 1997). Counterfactual thinking is conditioned by the availability of alternative options and opportunities accordingly. When an individual does not have any opportunities or opportunities imply inevitable negative consequences, the individual either mitigates or terminates the feeling of regret through attitude change, thus maintaining behaviour. In contrast, alternative decisions entailing a positive outcome facilitate the feeling of regret (Roese and Summerville, 2005) and predict switching behaviour (Lee and Lee, 2012). Feeling regret often results in corrective actions, a change of decision and behaviour, such as switching service providers if they fail to meet service requirements (Zeelenberg and Pieters, 1999). For example, a scenario-based experiment found that regretful decisions positively affect the intention to discontinue the use of technology and negatively affect satisfaction (Davvetas and Diamantopoulos, 2017). Regret experienced after the appraisal of service performance positively correlates with switching behaviour (Mattila and Ro, 2008, Sánchez-García and Currás-Pérez, 2011). Regret is a stronger predictor of behaviour modification when individuals compare the actual outcomes with better alternatives (Roese and Morrison, 2009). In addition, prior research provides evidence on the correlation between information-seeking and regret. It is suggested that the exposure to information about alternatives increases experienced regret (Keaveney et al., 2007), which potentially leads to a higher dissonance and the motivation to avert behaviour in order to reduce dissonance. Hence, this study proposes that:

- ***H5:*** *Feeling regret negatively affects a) attitude change and b) consonant information search, and positively affects c) behaviour change*

#### Satisfaction with Technology Performance and Perceived Wellbeing

According to Cognitive Dissonance Theory, the behaviour that arouses dissonance is associated with a negative affective state (e.g. dissatisfaction). In conditions when dissonance is aroused, satisfaction with the behaviour can be achieved if the psychological discomfort caused by disconfirmed expectations is eliminated. That happens by reducing the discrepancy between prior expectations and perceived performance using one of the dissonance reduction strategies (Shahin Sharifi and Rahim Esfidani, 2014, Dutta and Biswas, 2005, Festinger, 1962). Although all three dissonance reduction mechanisms (attitude change, consonant information search and behaviour change) reduce psychological tension, they trigger different levels of satisfaction with the behaviour causing dissonance. Specifically, attitude change and consonant information search refer to the cognitive dissonance reduction mechanisms that change the cognition (i.e. reinforcing positive beliefs about the behaviour), thus encouraging individuals to carry on the behaviour that initially caused dissonance (Harmon-Jones and Mills, 2019). By changing attitude and seeking consonant information, users increase the likelihood of experiencing satisfaction and perceived wellbeing (Festinger, 1962). In contrast, behaviour change reduces the psychological tension by eliminating the source causing dissonance (i.e. behaviour). That means that although the psychological tension is eliminated, the individual stays dissatisfied with the behaviour (Festinger, 1962). Such a theoretical explanation of the relationship between behaviour change and dissatisfaction is different from the stream of research which focuses on the relationship between disconfirmation – dissatisfaction – switching behaviour (Fan and Suh, 2014, Zhang et al., 2016, Lu et al., 2012, Nam et al., 2018) and overlooks the role of dissonance and dissonance reduction strategies. In this research, given the established dissonant state, withdrawal behaviour is one of the measures that people employ before evaluating satisfaction. The supporting arguments can be drawn from prior research, which found that users who are more committed to the behaviour are more likely to view the selected choice favourably and in turn experience higher satisfaction (Brehm and Cohen, 1962). For example, when individuals are engaged in interactive reflection on the behaviour, they change their cognition by strengthening their positive attitude to the behaviour and improving self-perception (e.g. self-confidence, self-awareness and self-knowledge) (Jones and Oswick, 2007). Sparks et al. (2012) examined a correlation between personality traits, reduction strategies and perceived satisfaction. They found that people who tend to maximise outcomes (maximisers) tend to withdraw behaviour, which results in less satisfaction. In contrast, non-maximisers tend to change the attitude towards the choice and perceive a stronger level of satisfaction. The study by Vroom and Deci (1971) provides evidence about the positive effect of the cognitive adjustment on satisfaction. The findings of the research postulated that when people do not engage in dissonance reduction through the change of cognition following the perception of discrepancy between expectation and the actual outcome, they show stronger dissatisfaction (Vroom and Deci, 1971). Given that satisfaction is a predictor of perceived wellbeing (Lee et al., 2002), discontinuous behaviour can be negatively associated with perceived wellbeing. Hence, the following hypotheses are put forward in relation to the cognitive dissonance coping mechanisms users may deploy:

- ***H6:*** *Attitude change has a positive effect on a) perceived wellbeing and b) satisfaction with technology performance*
- ***H7:*** *Consonant information seeking has a positive effect on a) perceived wellbeing and b) satisfaction with technology performance*
- ***H8:*** *Behaviour change has a negative effect on a) perceived wellbeing and b) satisfaction with technology performance*

**Fig. 2:**
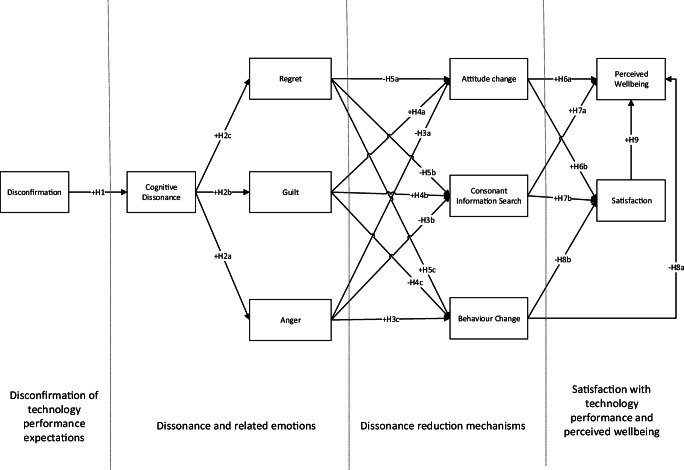
Research Model

Perceived wellbeing is a perceived impact on important life domains, which underpins the evaluation of the overall quality of life (El Hedhli et al., 2013). Perceived wellbeing reflects experiences with consumer goods and services (Lee et al., 2002). It is the result of satisfaction with the acquisition, consumption, possession and disposition of a product or service. Satisfaction in the consumer life domain has a spill over effect on other life domains (Lee et al., 2002). Wellbeing captures the cumulative satisfaction with the product and the positive experience that it has on user life, social life, leisure life and community life (El Hedhli et al., 2013). In other words, wellbeing is predicted by the satisfaction experiences, such as family relationships, the status in society, material possessions and education (Lee et al., 2002). This is of particular importance to the empirical setting of this work, namely smart homes. Smart homes aim to deliver individual and societal benefits by assisting in daily routines, delivering comfort, decreasing natural resource consumption (energy and water) and in turn reducing utility bills (Marikyan et al., 2019). Given that the aim of technology is to satisfy users’ needs, which tackle different aspects of life, a strong perception of fulfilled needs can contribute to user perceived wellbeing. The hypothesised relationships in the research model are provided in Fig. 2.

- ***H9:*** *Satisfaction with technology performance has a positive effect on perceived wellbeing*

## Methodology

### Data Collection and Sample

A survey was distributed through a research crowdsourcing platform to smart home technology users. A sample of smart home users was considered appropriate for the study. The adoption of smart home technologies reflects the digitalisation processes facilitating a sustainable lifestyle (GhaffarianHoseini et al., 2013). Since smart home systems promise social, economic and environmental benefits, users form high expectations about smart home performance (Marikyan et al., 2019). High expectations are usually difficult to confirm (Dwivedi et al., 2019), as evidenced by findings showing that the outcomes of smart home utilisation sometimes do not match up to the declared benefits (Hargreaves et al., 2018, Nicholls et al., 2017, Strengers and Maller, 2011). Given that the expectations-perception gap contributes to dissonance arousal (Venkatesh and Goyal, 2010), the focus on smart home users made it possible to examine emotions associated with dissonance and dissonance reduction mechanisms. The selection of the sample was conducted in two steps. The first step was to set the criteria for selecting respondents who used or had formerly used any smart home technology. This study did not focus on a specific device or system but rather aimed to recruit users of different types of smart home technologies (i.e. visual assistant, smart home security, smart alarms or leak sensors, smart lighting, smart plugs/switches, smart thermostat, smart home camera, smart vacuum cleaner, smart lock, smart kitchen, smart tag and smart entertainment systems) to have wider implications of the findings. Secondly, to be eligible to participate in the survey, the selected smart home users had to have a negative experience (e.g. problems with installation or facing privacy and security risks) with smart home technology. To verify that respondents had issues with the technologies, they indicated the type of negative incident that they had experienced by selecting it among a predefined list or b) typed the nature of the incident if this was not already included in the list. Out of 800 initially distributed questionnaires, 387 responses passed the filtering question and were valid for further analysis. The number of responses was deemed appropriate for running structural equation modelling (Hair, 2014). Table 1 presents the profile of the final sample of respondents. The profile includes information about the socio-demographic characteristics of the respondents (age, gender, education, marital status), the nature of the negative incidents, the type of utilised smart home technologies and usage patterns, measured by the length of technology usage and the perceived level of expertise. Perceived level of expertise was assessed by the multi-item scale developed by Mitchell and Dacin (1996). To categorise the sample into groups with low and high perceived expertise, the values of all the items were computed and converted into a dichotomous variable (1 = low expertise, 2 = high expertise) using a two-step cluster analysis in SPSS. The sample characteristics were collected to illustrate the sample and the context of the study in terms of the profile of smart home users, the types of negative incidents encountered, and the types of technology used. The information, such as the type of incidents and usage patterns, was useful for interpreting the relationships between the constructs.

**Table 1:** The profile of the respondents

**Demographic Characteristic**	**Type**	**Frequency (*n*** **= 387)**	**Percentage**
**Age**	18 to 24 years	111	28.7
	25 to 34 years	154	39.8
	35 to 44 years	80	20.7
	45 to 54 years	29	7.5
	55 to 64 years	11	2.8
	Age 65 or older	2	0.5
**Gender**	Male	186	48.1
	Female	185	47.8
	Other	16	4.1
**Education**	Completed some high school	27	7
	Completed some college (AS-A-Levels)	116	30
	Bachelor’s degree	156	40.3
	Master’s degree	72	18.6
	Ph.D.	6	1.6
	Other advanced degree beyond a Master’s degree	10	2.6
**Income**	Less than $25,000	89	23
	$25,000 to $ 34,999	78	20.2
	$35,000 to $ 49,999	70	18.1
	$50,000 to $ 74,999	64	16.5
	$75,000 to $99,999	48	12.4
	$100,000 to $149,999	26	6.7
	$150,000 to $199,999	7	1.8
	$200,000 or more	5	1.3
**Marital Status**	Single	221	57.1
	Married	142	36.7
	Separated	6	1.6
	Widowed	4	1
	Divorced	14	3.6
**Negative Experiences**	Technical issues during installation	100	25.8
	Technical issues during usage	119	30.7
	Ease of use	95	24.5
	Financial costs	16	4.1
	Privacy and security issues	26	6.7
	Other factors	31	8
**Smart Home Technology**	Visual assistant	289	77
	Smart home security	174	45
	Smart alarms or leak sensors	153	39.5
	Smart lighting	241	62.3
	Smart plugs/switches	216	55.8
	Smart thermostat	131	33.9
	Smart home camera	147	38
	Smart vacuum cleaner	113	29.2
	Smart lock	68	17.6
	Smart kitchen	92	23.8
	Smart tag	70	18.1
	Smart entertainment systems	216	55.8
**Subjective Expertise**	Low perceived expertise	138	35.7
	High perceived expertise	249	64.3
**Length of usage**	More than 10 years	11	2.8
	7–10 years	27	7
	4–6 years	141	36.5
	2–3 years	189	48.8
	Around 1 year	19	4.9

### Measurements

The questionnaire consisted of ten multi-item scales validated by prior studies (Table 2). Respondents were asked to answer questions by referring to their own specific incident when smart home technology did not perform as expected, which was captured at the beginning of the questionnaire. Items were measured by a 7-point Likert scale ranging between “1 - strongly disagree” to “7 – strongly agree”.

**Table 2:** Measurement items of constructs

**Measurement Item**	**Loading**	**α**
**Disconfirmation** Bhattacherjee and Premkumar (2004) **When compared to my initial expectations, smart home technologies involved in that incident...**		0.927
*increased my productivity when undertaking household tasks*	0.927	
*enhanced my effectiveness to undertake household tasks*	0.938	
*were useful in my daily routine at home when undertaking household tasks*	0.847	
**Cognitive Dissonance: Wisdom of Purchase** Sweeney et al. (2000) **Considering the instance where smart home technologies did not work as expected...**		0.906
*I wondered if I really needed those technologies*	0.719	
*I wondered whether I should have bought something else*	0.876	
*I wondered if I had made the right choice*	0.902	
*I wondered if I had done the right thing in buying those technologies*	0.877	
**Anger** Harmon-Jones et al. (2004) **After using smart home technologies in that incident, I felt...**		0.893
*Angry*	0.793	
*Agitated*	0.778	
*Irritated*	0.889	
*Frustrated*	0.843	
**Guilt** Coulter and Pinto (1995) **After using smart home technologies in that incident, I felt...**		0.901
*Accountable*	0.763	
*Guilty*	0.878	
*Ashamed*	0.864	
*Bad*	0.740	
*Irresponsible*	0.797	
**Regret** Tsiros and Mittal (2000) **After using smart home technologies in that incident, I felt...**		0.928
*I feel sorry for purchasing smart home technologies*	0.895	
*I regret purchasing smart home technologies*	0.970	
*I should have purchased traditional technologies for home instead of smart home technologies*	0.843	
**Attitude Change** Tussyadiah et al. (2018) **After using smart home technologies in that incident...**		0.903
*My liking toward them has been...*	0.900	
*My preference toward them has been...*	0.934	
*My interest in them has been...*	0.822	
**Consonant Information Search** Keng and Liao (2009) **After using smart home technologies in that incident...**		0.823
*I searched for information supporting my original positive beliefs about those smart home technologies on the Internet, on TV, radio, in newspapers, magazines, or reports*	0.806	
*I searched for information supporting my original positive beliefs about those smart home technologies through retail stores*	0.791	
*I asked people I know for positive comments about those smart home technologies*	0.738	
**Behaviour Change** Cho (2015), Chen et al. (2019) and Maier et al. (2015) **After using smart home technologies in that incident...**		0.872
*I temporarily stopped using them at home*	0.850	
*I used one or more alternatives to smart home technologies*	0.791	
*I used other technologies, instead of smart home technologies*	0.738	
**Satisfaction** McKinney et al. (2002) **Overall after using smart home technologies, I felt...**		0.953
*Satisfied*	0.950	
*Pleased*	0.956	
*Contented*	0.912	
*Delighted*	0.849	
*Will definitely recommend it to my friends*	0.837	
*Will definitely continue using it*	0.772	
**Well-being** El Hedhli et al. (2013) **Overall, smart home technologies...**		0.898
*Have satisfied my overall household needs*	0.819	
*Have played a very important role in my social well-being*	0.778	
*Have played a very important role in my leisure well-being*	0.846	
*Have played an important role in enhancing the quality of life in my household*	0.861	
**Subjective expertise** Mitchell and Dacin (1996) Please pick the answers that best apply to the statements:		0.908
*I am very familiar with smart home technologies*	0.877	
*I have a clear idea about which characteristics of smart home technologies are important in providing me maximum usage satisfaction*	0.808	
*I know a lot about smart home technologies*	0.908	
*I consider myself an expert about smart home technologies*	0.832	
*I have a lot of experience with smart home technologies*	0.868	

## Results

### Data Analysis

SPSS and Amos v.25 statistical tools were utilised for the analysis. SPSS v.25 was used to produce descriptive statistics. As the first step, we embarked on the confirmatory factor analysis using Amos v.25 to ensure that there were no reliability and validity issues. In line with the guidelines suggested by Hair (2014), confirmatory factor analysis demonstrated a satisfactory model fit (CFA: Model fit: χ2(657) = 1666.193, CMIN/DF = 2.536, CFI = 0.923, RMSEA = 0.063). Factor loading (>0.7), Cronbach’s α (>0.7), average variance extracted (AVE > 0.5) and construct reliability (C.R. > 0.7) were above the acceptable thresholds (Hair, 2014). The results of the convergent validity test, C.R. and AVE indices are presented in Table 3.

**Table 3:** Convergent validity test

	**C.R**	**AVE**	**1**	**2**	**3**	**4**	**5**	**6**	**7**	**8**	**9**	**10**
**Satisfaction**	0.955	0.781	**0.884**									
**Wellbeing**	0.898	0.687	0.811	**0.829**								
**Cognitive Dissonance**	0.911	0.72	−0.332	−0.281	**0.848**							
**Anger**	0.896	0.684	−0.282	−0.128	0.439	**0.827**						
**Guilt**	0.905	0.657	−0.129	−0.016	0.271	0.368	**0.811**					
**Regret**	0.931	0.819	−0.492	−0.421	0.617	0.489	0.527	**0.905**				
**Attitude Change**	0.911	0.774	0.646	0.626	−0.368	−0.327	−0.016	−0.430	**0.880**			
**Consonant Info Seeking**	0.823	0.608	0.110	0.229	0.202	0.156	0.313	0.185	0.247	**0.780**		
**Behaviour Change**	0.878	0.707	−0.514	−0.438	0.445	0.403	0.420	0.656	−0.413	0.292	**0.841**	
**Disconfirmation**	0.915	0.731	−0.463	−0.499	0.176	0.189	−0.010	0.274	−0.481	−0.247	0.308	**0.855**

*Notes: Diagonal figures represent the square root of the average variance extracted (AVE) and the figures below represent the between-constructs correlations*

### Path Analysis

The model fit indices were satisfactory (χ2(645) = 1874.935, CMIN/DF = 2.907, CFI = 0.904, RMSEA = 0.07), which made it possible to proceed with path analysis (Table 4). Out of twenty paths, four were non-significant. As hypothesised, the relationships between disconfirmation, cognitive dissonance and the three emotions were positive (H1 – H2c). When it came to the relationships between emotions and dissonance reduction strategies, there was a negative effect of anger on attitude change (H3a), a nonsignificant effect of anger on consonant information seeking (H3b) and a positive effect of anger on behaviour change (H3c). The effect of guilt on attitude change and consonant information seeking (H4a and H4b) was supported, but the relationship between guilt and behaviour change was not significant (H4c). The hypothesised relationships between regret and dissonance reduction strategies were confirmed (H5a and H5c), but the effect of regret on consonant information seeking was not supported (H5b). All relationships between dissonance reduction mechanisms and outcomes (i.e. satisfaction and wellbeing) were significant (H6a, H6b, H7a, H7b, H8b), except the effect of behaviour change on wellbeing (H8a). Hypothesis H9 was supported too, confirming a positive correlation between satisfaction and wellbeing.

**Table 4:** The results of the test of hypotheses

***H***	***Path***			***Coef.***	***t-test, sig***	***R***^***2***^
H1	Disconfirmation	➔	Cognitive Dissonance	0.182	(3.308***)	CD = 0.03
H2a	Cognitive Dissonance	➔	Anger	0.462	(7.735***)	Anger = 0.21
H2b	Cognitive Dissonance	➔	Guilt	0.308	(5.363***)	Guilt = 0.09
H2c	Cognitive Dissonance	➔	Regret	0.641	(11.604***)	Regret = 0.41
H3a	Anger	➔	Attitude Change	−0.193	(−3.527***)	Attitude Ch = 0.34
H3b	Anger	➔	Consonant Info. Seek	0.053	(0.804 ns)	Cons Info. Seek = 0.11
H3c	Anger	➔	Behaviour Change	0.104	(2.007*)	Behaviour Ch = 0.42
H4a	Guilt	➔	Attitude Change	0.296	(5.242***)	Satisfaction = 0.50
H4b	Guilt	➔	Consonant Info. Seek	0.313	(4.565***)	Wellbeing = 0.69
H4c	Guilt	➔	Behaviour Change	0.100	(1.884 ns)	
H5a	Regret	➔	Attitude Change	−0.483	(−8.118***)	
H5b	Regret	➔	Consonant Info. Seek	0.003	(0.039 ns)	
H5c	Regret	➔	Behaviour Change	0.583	(9.914***)	
H6a	Attitude Change	➔	Wellbeing	0.138	(2.672**)	
H6b	Attitude Change	➔	Satisfaction	0.517	(10.15***)	
H7a	Consonant Info. Seek	➔	Wellbeing	0.153	(3.549***)	
H7b	Consonant Info. Seek	➔	Satisfaction	0.096	(2.039*)	
H8a	Behaviour Change	➔	Wellbeing	−0.075	(−1.633 ns)	
H8b	Behaviour Change	➔	Satisfaction	−0.344	(−7.078***)	
H9	Satisfaction	➔	Wellbeing	0.673	(11.163***)	

## Discussion

### Disconfirmation of Technology Performance Expectations

The results of the analysis showed a significant and positive relationship between negative disconfirmation and dissonance (H1). The positive effect of disconfirmation on dissonance arousal was in line with the Cognitive Dissonance Theory (Festinger, 1962). Disconfirmation reflects the inconsistency between prior beliefs about technology performance and the actual perception of performance, thus inducing a psychological state of dissonance (Szajna and Scamell, 1993). Given the profile of the respondents, the majority of the sample considered that they had high expertise in technology (64.3%) and had actual utilisation experience of more than 2 years (95.1%). The higher the experience, the more critical is the ease of use factor (Al-Gahtani et al., 2007) and the easier is the use of more complex technologies (Beckers and Schmidt, 2003). The established relationship between disconfirmation and dissonance and the insight into the users’ characteristics suggests that performance issues were critical and the expectation-perception discrepancy could not be tolerated by users. The confirmed effect of negative disconfirmation on dissonance adds to the discussion raised by Park et al. (2015) and Park et al. (2012), who examined the consequences of inconsistency between the perception of pre-service and post-service performance. While they examined the discrepancy between the perception of services at different stages of technology use, the finding of this study provided evidence on the consequence of the incongruity between expectations and perceptions.

### Dissonance and Related Emotions

The positive effect of dissonance on anger, guilt and regret supported evidence from prior literature (Harmon-Jones, 2004, Harmon-Jones et al., 2017, Gosling et al., 2006, Gilovich et al., 1995b, Roese and Summerville, 2005). These findings made it possible to differentiate the effect of dissonance on each emotion independently, unlike the majority of prior studies, which focused on negative emotions in general (Jean Tsang, 2019, Gosling et al., 2006). The strength of the relationships demonstrated that the strongest feeling associated with dissonance was regret. The established effect of emotion suggests that individuals might have engaged in counterfactual thinking about a potential positive outcome of an alternative purchase decision (Croyle and Cooper, 1983). The effect of dissonance on anger was moderate. A significant relationship between dissonance and anger demonstrated that users did not feel in control and capable of using the technology the way they had initially expected (Harmon-Jones, 2004, Harmon-Jones et al., 2017). Given that anger is mostly experienced when people have low self-efficacy (Wilfong, 2006), the established relationship might suggest that weak technology performance was due to the personal inefficacy to perform the task. This explanation is also drawn from the profile of the respondents, who were mostly experienced users with high perceived expertise. This finding indicates that anger was not associated with a lack of experience with novel technology use, which could be accumulated along with the utilisation of technology. Rather, anger is related to the subjective evaluation of users’ incapability of dealing with the issue. The effect of dissonance on guilt was moderate too. Feeling guilt represents the state when people blame themselves for the violation of personal standards and norms (Harmon-Jones et al., 2017). The results suggest that improper technology performance might have disappointed users. They might have felt that they could not realise the potential of the technology they were fully in control of. Users might have had self-standards about technological self-efficacy, but they could not match up to those standards.

### Dissonance Reduction Mechanisms

The majority of the relationships between emotions and dissonance reduction strategies were significant. The findings supported the hypotheses that dissonance reduction strategies are predicted by emotions (Festinger, 1962). The differentiated effect of each emotion on reduction strategies was confirmed (Table 5). The correlation of emotions with different coping mechanisms demonstrated the complexity of negative emotions, dissimilarity in motivational direction (approach vs avoidance) and arousal strength (intensity in psychological response). When it came to the analysis of the role of each emotion in relation to a particular dissonance reduction strategy, the findings demonstrated that anger was negatively associated with attitude change and positively associated with behaviour change (H3a, H3c). This suggested that when users felt angry after experiencing weak technology performance, they tended to discontinue the use of those technologies, manifesting avoidance behaviour. This finding sheds light on the motivational role of anger, which has been disputed to date (Harmon-Jones, 2004, Carver, 2004, Smith and Lazarus, 1993, Harmon-Jones et al., 2017). Particularly, the findings contribute to the understanding of the approach and avoidance role of anger, depending on the context. Based on the descriptive statistics, the majority of incidents reported by respondents (67.4%) were rooted in the design of appliances (e.g. operation faults, integration issues, not robust security and privacy features) and only 24.5% of issues were due to low personal efficacy in utilising technology (i.e. ease of use). When an incident is the result of the appliance’s fault, anger motivates people to redress their experience by discontinuing behaviour (Donoghue and de Klerk, 2013). Hence, behaviour change served as a pro-active action representing the external means to cope with anger (Liang et al., 2019). Since anger is a very strong emotion, people tend to avoid future situations when they might be subjected to the same feeling. The insignificant effect of anger on consonant information seeking (H3b) showed that anger did not motivate people to balance the psychological state by adding consonant information to justify the choice.

**Table 5:** Relationships between emotions and dissonance reduction mechanisms

	**Anger**	**Guilt**	**Regret**
**Attitude Change**	–	+	–
**Consonant Information Seeking**	none	+	none
**Behaviour Change**	+	none	+

The relationships between guilt and dissonance reduction strategies confirmed a positive effect of guilt on attitude change and consonant information-seeking (H4a, H4b). The results are consistent with the perspective according to which guilt motivates approach behaviour (Kelman, 1979, Harmon-Jones et al., 2017, Ghingold, 1981a). Feeling guilt triggers the psychological coping mechanism, aimed at subduing the feeling of guilt. However, the results are inconsistent with the study by Turel (2016), who found that feeling guilt associated with the use of technology bringing intrinsic rewards results in discontinued use. Given that guilt undermines personal self-standards (Harmon-Jones et al., 2017), such as the belief in technological self-efficacy, this emotion predicts the change of cognition. The cognitive adjustment represents a coping mechanism reducing the feeling of inconsistency with one’s prior beliefs. By strengthening the positive attitude towards technology and seeking positive information about the technology, users justified the adoption and reduced dissonance. Although a negative effect of guilt on behaviour change was not confirmed, the lack of an established relationship may suggest that users feeling guilt tend not to discontinue the use of the technology. That means that the adoption of technology tackling environmental and social challenges is more likely to happen when weak smart home performance triggers a feeling of guilt.

Feeling regret had a moderate positive effect on behaviour change and a moderate negative effect on attitude change (H5a and H5c). The established effects were consistent with the findings of recent studies postulating that regret facilitates avoidance behaviour (Gilovich et al., 1995b, Davvetas and Diamantopoulos, 2017). In the context of the current research, regret is similar to anger in the way that these two emotions reflect a personal responsibility for the fault. However, regret is dissimilar from anger by the degree of counterfactual thinking that a regrettable decision implies (Connolly and Zeelenberg, 2002, Gilovich et al., 1995b). In line with the study by Roese and Summerville (2005), the established correlations between regret and reduction strategies demonstrated that self-blame and thinking about forgone alternatives decreased the value of the selected technology and demotivated continuous use. Given the effect size, out of all emotions, regret had the strongest power in regulating post-dissonance behaviour, suggesting that users gave a great deal of thought to opportunities that had been lost by refusing other alternative technologies. Similar to anger, the effect of regret on consonant information search was not supported (H5b), suggesting that there was no negative relationship between avoidance-directed behaviour and seeking consonant information. Given the effects of anger and regret, the negative experiences with smart homes overshadow their economic and social benefits and the positive implications for sustainability. That means that those emotions undermine the long-term utilisation of smart homes and the development of an intelligent ecosystem fostering societal transformation towards a sustainable lifestyle.

### Satisfaction with Technology Performance and Perceived Wellbeing

The analysis of dissonance reduction outcomes demonstrated that all relationships except the one between behaviour change and subjective wellbeing (H8a) were supported. The confirmed paths from attitude change and consonant information-seeking to perceived wellbeing and satisfaction confirmed the assumption that the positive outcome of weak technology performance can be achieved by adjusting cognitions. Those relationships confirmed the assumption that the reduction/elimination of cognitive discrepancy and psychological tension (Festinger, 1962) contributes to satisfaction (Vroom and Deci, 1971) and potentially increases perceived wellbeing. The findings were consistent with prior literature, which found a positive correlation between the tendency to favour a selected choice and satisfaction (Brehm and Cohen, 1962). The negative effect of behaviour change on satisfaction was supported too. In line with the study by Sparks et al. (2012), the withdrawal of behaviour was negatively associated with satisfaction. The lack of commitment towards the behaviour decreases the favourable attitude towards that behaviour, which is reflected in low satisfaction (Brehm and Cohen, 1962). However, the negative effect of behaviour change on perceived well-being was not supported. The finding suggests that when users discontinue the use of technology, they do not evaluate the degree to which smart homes improve the overall quality of life. The positive effect of satisfaction on perceived wellbeing adds to the research postulating that subjective wellbeing can be explained as the result of satisfaction with the use of a product or services, having a spillover effect on consumer life domains (Lee et al., 2002). Given that 95.1% of the respondents had more than two-years of experience with smart home technologies, the evaluation of the effect on satisfaction and well-being is based on long-term technology utilisation. Overall, the above findings provide two main pieces of evidence that have not been explored in the literature before. First, the findings confirm that despite negative incidents, the utilisation of smart homes may enhance users’ perceived wellbeing. That finding is important for the literature discussing the societal impact of data-driven smart technologies (Gupta et al., 2018, Pappas et al., 2018). Second, evidence about the psychological and behavioural consequences of disconfirmation feeds into the likely scenarios in which weak technology performance may result in satisfaction and perceived well-being.

## Theoretical and Practical Contributions

The findings of the study contribute to the literature in three ways. First, the study adds to the literature adopting the expectation-disconfirmation paradigm, which postulated that satisfaction is the outcome of the utilisation of technology, when performance exceeds prior expectations (Hsieh et al., 2010, McKinney et al., 2002). The findings of this study provide a different perspective by confirming a positive outcome following a weak performance of the technology. In addition, the results of the study add to the discussion by illustrating complex psychological processes following the evaluation of technology performance, which has not been explored before. A new insight into the disconfirmation-satisfaction relationship was made possible by extending the use of the Cognitive Dissonance Theory. Prior research used cognitive dissonance to explain the discrepancy between expectation and performance underpinning satisfaction/dissatisfaction (Elkhani and Bakri, 2012, Olson and Dover, 1979). This study used the cognitive dissonance framework to explain the conditions under which users facilitate their positive attitude, affective state about the technology and continuous use.

Second, the study contributes to the cognitive dissonance literature by providing evidence on the relationship between three distinctive negative emotions and three strategies to reduce dissonance. This study adds to the discussion of the underlying mechanisms of individuals’ behaviour in dissonant situations, such as rationalisation of behaviour or adjustment of perceptions to expectations (Fineman, 1997, Walsh et al., 2016). It takes a further step and explains the interrelation of the emotional, cognitive and behavioural factors underpinning the reduction of dissonance. While prior literature examined negative emotions including anger, guilt and regret as a unidimensional construct (Jean Tsang, 2019, Gosling et al., 2006), this study tested the effect that each has on attitude change, consonant information seeking and behaviour change. The study breaks down the characteristics and dimensions of each emotion and distinguishes their motivational role in approach or avoidance behaviour. By doing this, the research theorised and confirmed the significant role of guilt in dissonance reduction through cognitive adjustments, which in turn leads to satisfaction and perceived wellbeing. The role of regret and anger was confirmed to be a predictor of behaviour change and dissatisfaction.

Third, the findings of the study contribute to the literature on the utilisation of innovative technology by providing evidence on the psychological factors affecting consumer experience with smart homes. The focus adopted by the study is different from other research, which has mostly examined the factors underpinning the adoption of innovative technologies (Manis and Choi, 2019, Rauschnabel et al., 2015, Pizzi et al., 2019). While prior literature examined the predictors of the decision and processes of innovative technology adoption (Rogers, 1995, Dang et al., 2017, Oni and Papazafeiropoulou, 2014, Sabi et al., 2018), this research has investigated the behaviour of users after the appraisal of technology performance. The results are important to the literature, because the utilisation of technology is contingent on the perception of technology performance, which is often undermined by high expectations when it comes to innovative technology (Dwivedi et al., 2019, Sun and Medaglia, 2019, Fan and Suh, 2014). Also, the findings make a contribution to the smart home literature specifically. Prior studies discussed the use of smart home technology, its benefits and the factors underpinning behavioural intention to use (Balta-Ozkan et al., 2013b, Yang et al., 2017), but none had examined how people utilise the technology following a negative performance. This study provides insights into the psychological and behavioural factors following the evaluation of the performance of the technology.

The study provides some practical implications too. The findings provide practitioners with a user’s perspective on the utilisation of technology following disconfirmed expectations. Based on the results, people might continue using technology and even report satisfaction with the technology, despite the issues that they might face during use. However, when technology performance induces a feeling of regret and anger, people might cope with dissonance by discontinuing the use of smart homes. Therefore, practitioners need to focus more on the channels through which they can receive customers’ feedback in order to improve the technology. This is crucial for a competitive market, which can make people switch to alternatives to smart home technologies. In addition, the established strong feeling of regret and the effect it plays in behaviour change indicates that there is a retrospective consideration of the alternatives involved. This reflection often ends up in a better evaluation of the alternatives compared to the purchased product. Given that in regretful decisions people do not try to justify the decision by a consonant information search, the post-factum communications with customers seems to be an ineffective tool in retaining customers. Therefore, the marketing and sale of innovative technology should encompass trustworthy and comprehensive information about technology services, functions and benefits in order to set realistic expectations. Finally, the reported feeling of anger and the following abandonment of technology indicate that people perceive the fault in technology performance to be irreversible. Practitioners need to investigate all possible instances of poor technology performance to change or eliminate the likelihood of the arousal of this emotion.

The findings of the study about the effect of emotions on approach and avoidance behaviour provide recommendations for the developers of health-oriented smart homes. Health-oriented smart homes integrate emotion recognition technologies based on face image processing to identify the patient’s health status (Mano et al., 2016). The findings of this study suggest that apart from emotions reflecting users’ physical health, the technology can also be used to capture the emotions while interacting with technology. The developers need to distinguish two types of recorded emotions based on the proximity of the technology to the user at the time of facial expression recognition. The algorithm can be used to sort and analyse those two types of data to increase the accuracy of the results and inferences. Given the effects of anger, guilt and regret on approach behaviour, the emotions expressed at the time of interaction with technologies enable developers to predict the likelihood of continuous use. Furthermore, the findings can be utilised to explore the aspects of the use of technology triggering negative emotions. That helps identify which areas need improvement and increase technology adoption in households. The development of home-based healthcare using smart technologies is especially important considering the pressure on healthcare systems posed by the growing ageing population (Kankanhalli et al., 2016) and in the reality of the spread of COVID-19.

Drawing on prior research (Pappas, 2018, Pappas et al., 2016) confirming that there are several ways through which consumers’ purchase intention can be shaped, the results of this study about the effect of emotions provide practical suggestions as to how to change consumer emotions and intention. User experience with ICT can result from the use of different solutions and services stimulating various emotions (Pappas, 2018). Given that those affective states may have distinctive paths to approaching behaviour (use intention and continuous use intention), the introduction of an add-on service may induce another type of emotion with different predictive power. The combination of emotions may shift the effect of anger or guilt towards a more positive outcome. Therefore, the results produced by this study and evidence from prior research (Pappas, 2018, Pappas et al., 2016) suggest that practitioners should develop solutions offering a range of customised services. These may induce positive emotion, such as happiness, attenuating the withdrawal effect of negative emotions.

On a larger scale, the study provides actionable insights into the behaviour of users that can be used to increase the adoption of smart homes and the growth of a data-rich ecosystem. The data generated about the behaviour of individuals may facilitate organisational decision making, provide answers to sustainability challenges and address social issues by developing sustainable solutions. The growth of such an ecosystem reflects the digital transformation towards sustainable societies. Specifically, the findings of the study have implications in the sphere of smart city development. Given that a smart home is an integral part of smart cities (Ismagilova et al., 2019), the findings can be helpful in securing a higher level of smart technologies embeddedness in the smart urban ecosystem. A higher adoption of smart home data-driven technologies may accelerate the sustainability effect, which is the goal of smart cities (Ismagilova et al., 2019, Kar et al., 2019). The data generated by smart homes can provide insights into the human-technology interaction and the communication between the stakeholders of the smart environment. The generated information may elucidate the mechanisms accelerating the sustainability impact of smart cities and the development of sustainable solutions.

## Conclusion and Future Research Suggestions

The study explored the outcome of the use of innovative technology in conditions when the performance of technology did not meet expectations based on a sample of smart home users. The research model theorised and confirmed that the disconfirmation of expectations can result in satisfaction and wellbeing when dissonance-induced emotions activate coping mechanisms aimed at reducing dissonance. The model established a positive correlation between dissonance, anger, regret and guilt. Distinctive effects of the three types of emotions on the reduction of cognitive dissonance through attitude change, consonant information-seeking and behaviour change were found. Finally, the effect of dissonance reduction through cognitive adjustment (consonant information seeking and attitude change) on satisfaction and perceived wellbeing was confirmed. These results illustrate the psychological and behavioural responses of individuals which may happen when technology does not perform as expected. The emotional profile of users indicates that the performance of technology makes people question the purchase decision and makes users think that nothing can be done to improve the use of technology. Those feelings are more likely to end up in switching the product for another alternative. However, when people think that by using technology they have transgressed their values, they try to justify their purchase decision, which is likely to contribute to continuous use, satisfaction and perceived wellbeing.

The study has some limitations. First, we used a cross-sectional approach to test the research model. Future studies could examine the relationship between cognitive dissonance, emotions and dissonance reduction longitudinally. A longitudinal approach would make it possible to observe the change of emotions and behaviour over time, thus increasing the accuracy of the results about the proposed relationships. Second, future research could test the moderation effect of personal factors, such as self-efficacy, perceived behavioural control or the tendency to outcome maximisation to receive a more precise picture about the contingency of coping mechanisms on individual characteristics. Third, the study uses self-reported data to infer the interrelationships between emotions, dissonance reduction and positive outcomes (satisfaction and wellbeing), which might be subjective. Future studies may collect physiological data to detect behaviours following dissonance arousal, in line with the prior study which explored the correlation between online content reviewing and purchasing behaviours using an eye-tracking system (Mikalef et al., 2020). For example, future research may investigate consonant information search by examining gazing transitions between consonant and dissonant information about the technology. In addition, the adoption of smart homes can be facilitated if smart home technologies start recording and analysing facial mimicry and gaze-based interaction like some mobile technologies do (Khamis et al., 2018). The recording of such data would make it possible to infer the patterns of technology exploitation and user experience, based on the correlation between individuals’ behaviour and eye movements. That would move forward future research on smart home adoption. Fourth, this research examined emotions using structural equation modelling, which explores the “net effects” of independent variables on dependent variables (Woodside, 2014). However, individuals’ reactions may be caused by multiple configurations of cognitive and affective factors (Pappas et al., 2016, Pappas et al., 2020). Hence, future research could use a methodological approach addressing complex, nonlinear and dynamic relationships between variables (Woodside, 2014), such as fsQCA, and could test the effect of emotions with a set of other cognitive or situational factors.
